# Protein expression profiling of nuclear membrane protein reveals potential biomarker of human hepatocellular carcinoma

**DOI:** 10.1186/1559-0275-10-6

**Published:** 2013-06-01

**Authors:** Rizma Khan, Saadia Zahid, Yu-Jui Yvonne Wan, Jameson Forster, A-Bashar Abdul Karim, Atta M Nawabi, Abid Azhar, M Ataur Rahman, Nikhat Ahmed

**Affiliations:** 1 Neurochemistry Research Unit Laboratory, Department of Biochemistry, University of Karachi, Karachi, Pakistan; 2 The University of Kansas Medical Center, Kansas City Lawrence, Kansas, USA; 3 The Karachi Institute of Biotechnology and Genetic Engineering, (KIBGE), University of Karachi, Karachi, Pakistan

**Keywords:** Hepatocellular carcinoma, Cytochrome b5A, Two dimensional gel electrophoresis, S-nitrosylation, Proteomics, ESI-Q-TOF MS/MS mass spectrometry

## Abstract

**Background:**

Complex molecular events lead to development and progression of liver cirrhosis to HCC. Differentially expressed nuclear membrane associated proteins are responsible for the functional and structural alteration during the progression from cirrhosis to carcinoma. Although alterations/ post translational modifications in protein expression have been extensively quantified, complementary analysis of nuclear membrane proteome changes have been limited. Deciphering the molecular mechanism that differentiate between normal and disease state may lead to identification of biomarkers for carcinoma.

**Results:**

Many proteins displayed differential expression when nuclear membrane proteome of hepatocellular carcinoma (HCC), fibrotic liver, and HepG2 cell line were assessed using 2-DE and ESI-Q-TOF MS/MS. From the down regulated set in HCC, we have identified for the first time a 15 KDa cytochrome b5A (CYB5A), ATP synthase subunit delta (ATPD) and Hemoglobin subunit beta (HBB) with 11, 5 and 22 peptide matches respectively. Furthermore, nitrosylation studies with S-nitrosocysteine followed by immunoblotting with anti SNO-cysteine demonstrated a novel and biologically relevant post translational modification of thiols of CYB5A in HCC specimens only. Immunofluorescence images demonstrated increased protein S-nitrosylation signals in the tumor cells and fibrotic region of HCC tissues. The two other nuclear membrane proteins which were only found to be nitrosylated in case of HCC were up regulated ATP synthase subunit beta (ATPB) and down regulated HBB. The decrease in expression of CYB5A in HCC suggests their possible role in disease progression. Further insight of the functional association of the identified proteins was obtained through KEGG/ REACTOME pathway analysis databases. String 8.3 interaction network shows strong interactions with proteins at high confidence score, which is helpful in characterization of functional abnormalities that may be a causative factor of liver pathology.

**Conclusion:**

These findings may have broader implications for understanding the mechanism of development of carcinoma. However, large scale studies will be required for further verification of their critical role in development and progression of HCC.

## Introduction

Hepatocellular carcinoma (HCC) is the third leading cause of cancer related deaths worldwide [1-4], increasing from 1.8 to 2.5 per 100,000 patients. Hepatitis B and C viral infections are well recognized underlying cause of chronic liver disease leading to HCC whereas dietary exposure to aflatoxin B1, alcoholic liver dysfunction and autoimmune hepatitis are also renowned risk factors [5-9]. The prognosis of HCC is dismal due to underlying cirrhosis as well as poor tumor response to chemotherapeutic regimens [10-12]. Opportunity for anti-cancer therapy in early stage is overlooked just because of the lack of effective biomarkers [13,14]. Complex molecular events lead to development and progression of liver cirrhosis to HCC. Deciphering the molecular mechanism that differentiates between normal and disease state may lead to identification of biomarkers for carcinoma [15,16]. Although alterations in protein expression have been extensively quantified during progression from cirrhosis to carcinoma, complementary analysis of nuclear membrane proteome changes has been limited [17].

HCC has been associated with elevated expression of inducible nitric oxide synthase (iNOS), and has been responsible for high-output production of nitric oxide (NO). Innate immune response and inflammation, NO is often highly increased at mRNA and protein levels in patients with chronic HBV and HCV [18-20], hemochromatosis and alcoholic cirrhosis [20] all of which cause predisposition to HCC [21]. Nitrosylation is essential and an important reversible post translational modification (PTM) of proteins [22-24]. It is a potential modulator of cellular processes important for tumorigenesis, apoptotic cell death and inhibition of DNA repair [25-27]. In addition to phosphorylation, DNA repair pathways are regulated at multiple levels by NO key components that depict an important role in pathogenesis of hepatocellular carcinoma [25,28-30].

Here we interrogate the differential proteome profiling in HCC tissues of clinically diagnosed HCC patients, fibrotic liver and HepG2 cell lines as controls. We explored HCC nuclear membrane CYB5A as down regulated and nitrosylated. The altered expression of CYB5A suggests that these proteins may be used as a novel prognostic factor and possibly an attractive target for HCC. CYB5A has been associated with essential cellular processes that include cytochrome P450 mediated metabolism of xenobiotics, drugs [31-34], and homeostasis of cholesterol and steroid hormone [35-37]. Involvement of CYB5A in methemoglobin to hemoglobin reduction in erythrocytes [38], and hydroxylation of N-acetyl-neuraminic acid is also observed [39]. Additionally, naturally existing fusion enzymes include mitochondrial flavocytochrome b_2_ (L-lactate dehydrogenase) [40], sulfite oxidase [41], the Δ ^5^ and Δ ^6^ -fatty acid desaturases (stearyl-CoA-desaturase) [42] and yeast inosi tolphosphorylceramide oxidase also contains CYB5A as a domain component [43].

## Results

### Differential assessment of HCC proteome

In an effort to profile differentially expressed proteomic alterations in HCV infected liver during cancer progression, we used 2-DE coupled with ESI-QTOF MS/MS to determine the relative levels of proteins across HCC and fibrotic liver.

Approximately 864 protein spots were detected on 2DE gels, out of which 76 protein spots exhibit differential expression in HCC as compared to fibrotic liver and HepG2 cell line. The quantity of each spot was normalized as a percentage of the total quantity of all gel spots. Differentially expressed proteins were defined as statistically significant on the basis of >1.5 fold up and down regulation in HCC patients compared with cell line or more changes in expression intensity (*p* < 0.05). Gel analysis was performed using Progenesis SameSpots v4.5 (Nonlinear Dynamic, UK). Each sample set (n = 6) was analyzed in 5 independent mass spectrometer runs.

**Figure 1:**
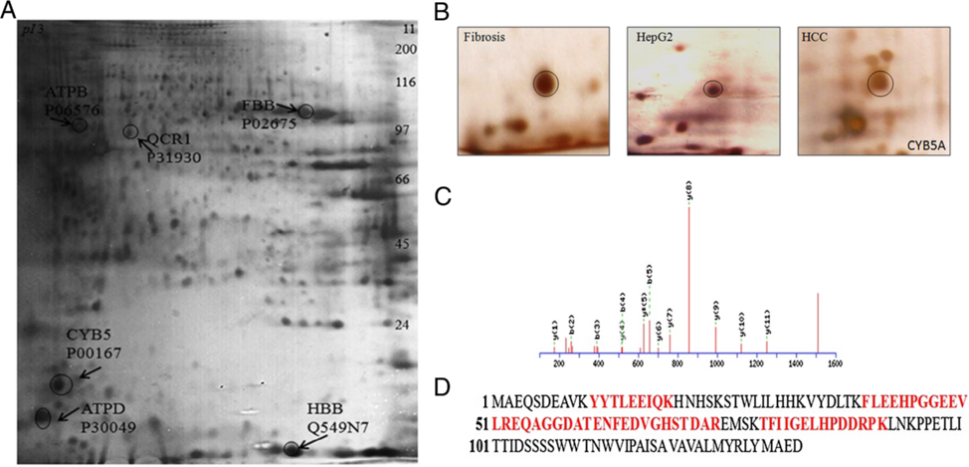
**Targeting CYB5A as potential biomarker. (A):** in this representative expression profile of significantly up (ATPB, FIBB, and QCR1) and down regulated (CYB5A, ATPD and HBB) proteins in HCC compared with fibrotic liver and HepG2 cell line. Focused on IPG strips pH 3–11 NL (11 cm) and separated on 12.5% gels followed by silver staining. Gel analysis was performed by using Progenesis SameSpots v4.5 (Nonlinear Dynamic, UK). The statistical analysis using Student t-test was performed and on the basis of normalized volumes spots were selected with p-values (< 0.05). (**B):** Representative protein spot of CYB5A (indicated by circle), showing differential expression between HCC, fibrotic liver and HepG2 cell line. The protein spots were analyzed by using ESI-QTOF MS/MS. (**C):** Representative MS/MS spectrum and peptide sequence of CYB5A identified by one or more high scoring peptides were considered to be true matches or with extensive homology (p < 0.05). b and y represent b ions and y ions respectively, generated during peptide fragmentation in MS/MS. (**D):** Amino acid sequence of CYB5A, matched peptides were expressed in red type and underline.

**Figure 2:**
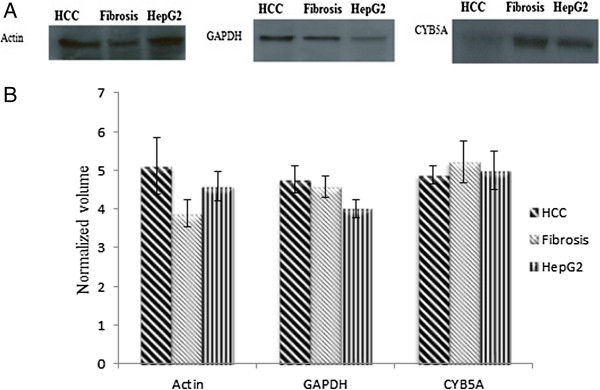
**Immunoblot analysis confirms changes in protein expression of CYB5A. (A):** representative western blot analysis of CYB5A in HCC, fibrotic liver and HepG2 cell line. For this, 100μg of nuclear membrane samples were loaded onto a 12% SDS-PAGE. Expression level was analyzed using the primary antibody CYB5A (1:1000 dilution) and horse raddish peroxidase (HRP) - conjugated secondary antibody (1: 5000 dilution). Β-actin and GAPDH were used as a loading control. **(B):** representative graphical expression of validated proteins by western blotting. Digital images were taken by gel documentation system (Bio-Rad). Quantification and intensity measurement of protein bands were analyzed by Quantity One gel analysis software (Bio-Rad). Statistical significance (p- value > 0.05) was calculated using SPSS statistics version 17.

The data revealed, for the first time, additional proteins that were dysregulated in HCC compared with fibrotic liver and HepG2 cell line. These include significantly elevated levels of ATPB, fibrinogen beta chain (FIBB), and cytochrome b-c1 complex subunit 1(QCR1). Included among the proteins that were down-regulated and not previously reported were CYB5A, ATPD and HBB well represented in Figure 1A. The protein spots were analyzed by using ESI-QTOF MS/MS. Total of six proteins along with accession no. obtained from SWISS/Prot and sequence coverage (%) refers to the percentage of protein sequence coverage, determined by number of matched peptides, and their functions were described in Table 1, Additional file 1. Due to the functional significance of CYB5A, we focused on the decreased expression of CYB5A observed in HCC as compared to fibrotic liver. The protein expression along with MS/MS spectra and matched sequence are shown in Figure 1(B-D). In order to assess the validity of data, we examined the differentially expressed CYB5A protein by western blot. The expression of CYB5A was seen to be relatively down regulated in HCC as compared to HepG2 cell line and fibrotic liver Figure 2(A-B).

**Table 1:** Differentially expressed HCC, fibrotic liver nuclear membrane proteins and HepG2 cell line

**Acc**	**Name**	**Abb**	**Thr. pI; mass**	**Score**	**Peptide matched**	**Cellular compartment**	**PTM**	**%age cov**	**Mean normalized volume fibrosis HCC /HepG2**		**Ratio**	**Anova (P)**	**Fold change**
						CF(1), Membrane							
**P30049**	ATP synthase subunit delta	ATPD	5.38; 17	58	5	mitochondrion	Isopeptide bond	17%	0.02536	0.00704	0.277	0.02	3.6
						Mitochondrion	Ubl conjugation						
						inner membrane							
**P02675**	Fibrinogen beta chain	FIBB	8.54; 55	1009	27	Secreted	Disulfide bond	41%	0.03364	0.01628	0.483	0.05	2.1
							Glycoprotein						
							Pyrrolidone						
							carboxylic acid						
**P06576**	ATP synthase subunit beta	ATPB	5.26; 56	670	18	CF(1), Membrane	Acetylation	29%	0.03448	0.00913	0.264	0.04	3.7
						mitochondrion	Phosphoprotein						
						Mitochondrion inner membrane							
**Q549N7**	Hemoglobin subunit beta	HBB	6.75;15	484	22	Heptoglobin-	Acetylation	61%	0.22697	0.11803	0.520	0.01	1.9
						hemoglobin	Glycation						
						complex	Glycoprotein						
							Phosphoprotein						
							S-nitrosylation						
**P00167**	Cytochrome b5 OS	CYB5A	4.88; 15	176	11	Cytoplasmic	Acetylation	42%	0.01948	0.05883	3.020	0.04	3.0
						Endoplasmic							
						reticulum							
						Membrane							
						Microsome							
**P31930**	Cytochrome b-c1 complex subunit 1	QCR1	5.94; 52	149	10	Mitochondrion	Acetylation	15%	0.01607	0.00505	0.314	0.05	3.1
						inner membrane	Phosphoprotein						

Accession no. is obtained from SWISS/Prot and percent coverage refers to the percentage of protein sequence coverage, determined by number of matched peptides. Significance was calculated from the Progenesis SameSpots v4.5 (Nonlinear Dynamic, UK), generated statistical analysis. A value of p < 0.05 was considered statistically significant. Functional classes were determined by searching the UniProt database (http://www.uniprot.org).

The proteins were separated on 2DE gels and identified by ESI-QTOF MS/MS.

### CYB5A is an S-Nitrosylated protein

**Figure 3:**
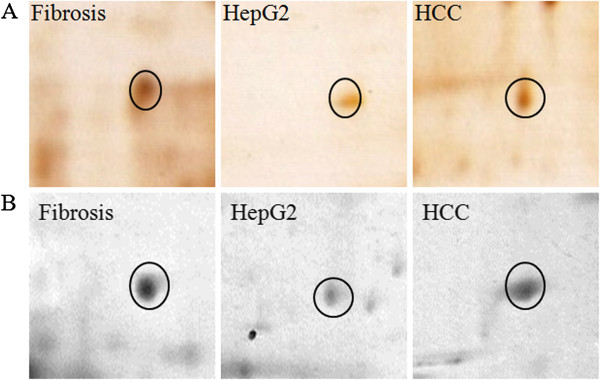
**Antibody based identification of CYB5A as S-nitrosylated protein. (A):** 2DE Spot representative differential expression level of CYB5A as S-nitrosylated protein in HCC compared with respective fibrotic liver and HepG2 cell line. The proteins were immuno-precipitated with anti SNO-Cys antibody, focused on IPG strips (pI 3-11NL; 7 cm) and separated on 12.5% gel followed by silver staining. **(B):** Representative spots of CYB5A immuno-precipitated with anti SNO-Cys antibody. 100 μg proteins were focused using pI 3-11NL (7 cm) IPG strips and separated on 12.5% gel followed by western blotting described in material and methods. Densitrometric analysis was performed using Progenesis SameSpots v4.5 (Nonlinear Dynamic, UK).

CYB5A a key determinant of our study was observed to be differentially S-nitrosylated in HCC, fibrotic liver and even HepG2 cell lines. An increased intensity of S- nitrosylation in the fibrotic tissue is revealed by 2-DE-IP and western blot analysis, relatively low intensity in HCC and very low in case of cell lines Figure 3(A-B) respectively.

### Immunohistolocalization of CYB5A

**Figure 4:**
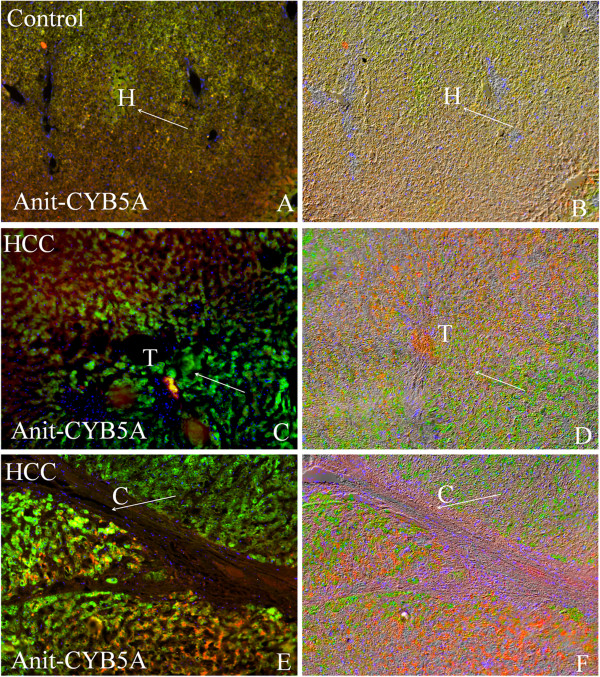
**Immunofluorescence analysis of CYB5A in HCC.** The IHC image of CYB5A staining in control (**A**) and hepatocellular carcinoma (**C** &**E**) formalin fixed paraffin embedded (FFPE) section. The section was pre-treated using heat mediated antigen retrieval with sodium citrate buffer for 30min. The section was then incubated with CYB5A, 5 μg/ml, for 1hr at 40^o^C. The secondary antibody (green) was Alexa Fluor® 488 goat anti-mouse IgG (H+L) used at a 1/1000 dilution for 30min at 40^o^C. DAPI was used to stain the cell nuclei (blue) at a concentration of 1.43 μM. **(B, D & F):** combination of DIC images and IHC of control and HCC respectively, work done by Adobe Photoshop CS2. (C=Portal tract; H=Normal hepatocytes; T=Malignant hepatocytes; Original magnification= 20X). IHC: Immunohistolocalization.

IHC analysis of the CYB5A shows significant expression in malignant hepatocytes. However, no expression was observed in portal vein Figure 4(A-B). We also studied disseminated intravascular coagulation (DIC) images, created with Adobe Photoshop CS2 images that exposed the histology and morphology of cells on the same sections. Both immunofluorescence and DIC images were stacked in Figure 4(B). All information related to microscope and camera setting is provided in the supplementary data (Additional file 2).

### Immunohistolocalization of S-nitrosylated protein

**Figure 5:**
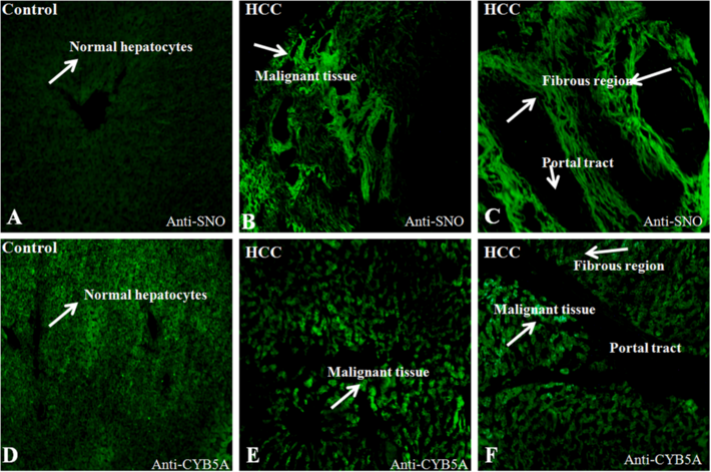
**Evidence of S-nitrosylation in HCC: Increased S-nitrosylation is evident in the fibrous portion and tumor cells in HCC.** Anti SNO-Cys antibody was used to reveal the distribution of S-nitrosylation signals in the HCC and controls. Significant increase CYB5A is also evident in both fibrous and tumor cells similar to S-nitrosylation. Figure **A** &**D** represents control liver on the other side **B, C, E, F** explained strong S- nitrosylation signals in HCC.

Increased S-nitrosylation signal was observed in tumor and fibrous region of HCC tissue as compared to controls Figure 5(A, B & C). A significant increase in S-nitrosylation intensity of CYB5A is also evident in this regions (Tumor and Fibrosis), revealed by immunofluorescence images Figure 5(C, D & E). The results presents a massive disruption of lobular manner, portal tract expansion with inflammatory cells in the sinusoids, lymphoid aggregate and hepatocellular apoptosis in the regions with hyper S-nitrosylation signals. The histological and morphological defects were assessed in these regions using Hemotoxylin and Eosin (H&E) staining prior to the immunoflorescence analysis.

### The predicted functional association network of CYB5A

**Figure 6:**
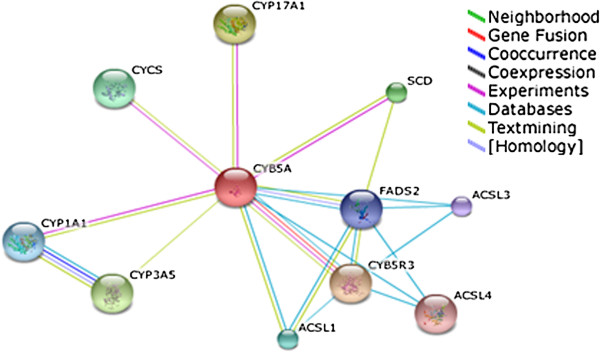
**Functional association network of CYB5A.** High-confidence protein-protein interaction network of identified nuclear membrane proteins derived from the STRING database (http://string-db.org/). Each protein is represented as a node with edged interactions. (CYB4R3: cytochrome b5 reductase 3; CYP17A1: Cytochrome P450 family 17 subfamily A polypeptide 1; CYP3A5: cytochrome P450 family 3, subfamily A, polypeptide 5; FADS2: fatty acid desaturase 2; SCD: stearoyl-CoA desaturase; ACSL1, L2, L3 and L4: acyl-CoA synthetase, long chain family member 1, 2, 3 and 4 respectively).

The functional association network of the identified proteins was generated through protein interaction network STRING 8.3. The interaction patterns will be helpful to have a better understanding of the protein functional activities. As the identified proteins are involved in various cellular/metabolic pathways the close interaction pattern, evident with the high confidence score > 0.7 are helpful to explicate disease related consequences due to structural and functional perturbations of the expressed protein Figure 6. CYB5A is a membrane bound hemoprotein strongly interacting with its class member CYB4R3 (cytochrome b5 reductase 3), CYP17A1 (Cytochrome P450 family 17, subfamily A, polypeptide 1), CYP3A5 (Cytochrome P450 family 3, subfamily A, polypeptide 5). Possible strong interaction with FADS2 (fatty acid desaturase 2), SCD (stearoyl-CoA desaturase), ACSL1, L2, L3 and L4 (acyl-CoA synthetase, long chain family member 1, 2, 3and 4 respectively) are also observed Figure 6.

### Pathway enrichment analysis related to HCC pathology

Having validated our Mass spectrometry result of CYB5A, we delineated the biological function associated with this elevated protein alterations based on the known protein functions according to SWISS-PROT (http://www.expasy.org), classifications provided in the KEGG database (http://www.genome.jp/kegg), GO annotation (http://www.geneontology.org), Reactome, Uniprot, ENSEMBL, PINC analysis pathway and Panther classification system. This process allowed us to identify pathways that are deregulated during HCC development and progression. This exercise revealed the involvment of CYB5A in vitamin C (ascorbate) metabolism, metabolism of water-soluble vitamins and cofactors, metabolism of vitamins and cofactors, transport, L-ascorbic acid metabolic process, electron transport chain, small molecule metabolic process, aldo-keto reductase (NADP) activity, amino sugar and nucleotide sugar metabolism http://www.reactome.org.

## Discussion

The current study identifies novel nuclear membrane biomarkers for HCC. Out of 76 differentially expressed proteins, six proteins ATPD, FIBB, ATPB, HBB, CYB5A, and QCR1 were quantified across the specimens and delineated into liver fibrosis and carcinoma specific proteins. CYB5A trapped our main focused not only because of lack of information regarding its sub cellular location and down regulation in nuclear membrane of HCC compared to fibrotic liver and HepG2 cell lines, but also because of S-nitrosylation of this protein. Interestingly, CYB5A is characterized by the presence of 3 well-defined phosphorylation sites as demonstrated by the insilico analysis (NetPhosK 1.0). It is likely that incomplete phosphorylation, (may be due to nitrosylation) i.e. not all monomers are phosphorylated, may interfere with protein function. Emerging data suggests that CYB5A and other nuclear proteins are involved in HCC progression [44-46]. Further study will be required to clarify how phosphorylation and nitrosylation influence HCC and the consequent expression of proteins.

Validation by western blotting, immunoprecipitation and immunohistochemical studies revealed the characterized pattern in malignant cells and nuclei but was absent in portal tract. These results allow us to propose sub cellular localization of CYB5A as down regulated nuclear membrane protein with a mechanism proposed in various pathophysiological conditions as aberrant S-nitrosylation, caused by altered NO production. So we can say that, decreased expression could leave the liver predisposed to the oncogenic effects of nitrosative stress. Additional study is needed to determine whether NO production induces post-translational modifications of protein and modulates protein function in HCC cell. However, due to the absence of cysteine residue in the CYB5A protein sequence, we propose that the other thiol groups are nitrosylated in the studied HCC specimens.

Understanding towards the disease progression requires complete knowledge interrelated to different pathways and their involvement with the altered proteins.

We conducted STRING 8.3 generated patterns which demonstrated direct interaction of these metabolic proteins with other associated proteins, CYB4R3, CYP17A1, CYP3A5 FADS2 SCD and ACSL1, has an essential role in the oxidative reaction, anabolic metabolism of fat and steroids, catabolism of xenobiotics and endogenous metabolism [47]. It also has a rather complex interaction with Cytochrome P450 and stimulates its related metabolism [33,48,49].

Insilico studies revealed the role of CYB5A in aldo-keto reductase (NADP) activity, cytochrome-c oxidase activity, enzyme binding, heme binding as reflected by the KEGG/REACTOME pathway analysis. Its involvement in respiratory electron transport chain, lipid metabolic pathway was further elaborated by Panther classification (http://www.panther.com). Several lines of evidence provide insight into potential mechanism by which CYB5A may affect HCC progression and associated pathological condition [25,27]. In our case, the presence of 15 -KDa CYB5A fragment in the HCC specimens compared to fibrotic liver and HepG2 cell lines clearly indicates the possible role of this protein in cancer progression. The novel discovery of a membrane protein CYB5A down regulation and its localization coupled with the observation of intense nitrosylation signals in the regional architecture of cirrhotic tissue strongly suggests a major role of CYB5A in the physiology and pathophysiology of HCC.

## Conclusion

Our study suggests that nuclear membrane proteins may have a role in progression from fibrosis to liver carcinogenesis. The molecular mechanisms controlling CYB5A expression and function are of paramount importance. Moreover, target protein activation may be influenced by nitrosylation, might represent a mechanism insuring that a cirrhotic tissue development process was underway where CYB5A and other different proteins can be applied to post translation regulation. In this regard recent studies are beginning to establish biochemical connections between different proteins and cell signaling molecules. Further work must be performed to determine whether these changes are essential and appropriate for HCC diagnosis and prognosis.

## Material and methods

### Tissue procurement

The Institutional Review Board of University of Kansas Medical Center (USA) and University of Karachi, Pakistan approved this study on discovery of nuclear membrane proteome of liver cancer. Well characterized HCV-associated HCC liver tissues (n = 50) were by Kansas Medical Center (KUMC) Liver Bank, USA and Karachi hospitals [male 25 age in mean + SD 53 ± 7.06); female = 25 (51.1 ± 3.35)] while the fibrotic liver tissue [n = 65; male = 45 (49.4 ± 5.8) & female = 20 (47.4 ± 5.4)] were collected only from Karachi, Pakistan with confirmed clinical and genotype report. Tissues were snap-frozen in dry ice and stored at −80°C.

### Extraction of protein from HCV infected liver

For protein extraction, samples [HCC (n = 50) fibrotic liver (n = 65)] were immersed into PBS (phosphate buffer saline) buffer and washed. Homogenized in adequate volume of homogenization medium (0.25 M sucrose and 10 mM HEPES, pH 7.5), and centrifuged at 1000xg for 10 min. The pellet and supernatant were collected and the supernatant resuspended with buffer A (0.3 M sucrose, 50 mM tris, and 3 mM MgCl_2_, pH 7.5) and buffer B (1.98 M sucrose, 50 mM tris, 1 mM MgCl2, pH 7.5), centrifuged at 70,000xg for 90 min (Optima L-100 XP, Beckman Coulter, USA).Protease (Cocktail tablet, Roche, Germany) and phosphatase inhibitors (0.2 mM sodium orthovanidate Na_3_Vo_4_ and 1 mM sodium fluoride NaF) were added at every step [17]. The pellet containing nuclei fraction was collected and resuspended in lysis buffer (7 M urea, 2 M thiourea, 4% CHAPS, 30 mM tris-Cl, pH 8.5, protease inhibitor mixture), stored at −80°C. Total protein content was measured using the Bradford protein assay.

### Cell culture and cell lysis

HepG2 cell line grown in Dulbecco’s modified Eagle medium (DMEM) containing 10% fetal calf serum (FCS) was used. Briefly, cells were rinsed with DMEM and removed from the flask by incubating with a solution containing trypsin (0.5 g/l) and EDTA (0.2 g/l). The cells were detached from the surface of the flask by squirting the solution onto the cells. The suspension was centrifuged at 1000xg for 5 min and the cells washed with DMEM without FCS. After centrifugation and removal of DMEM, cells were mixed and solubilized. The cells were washed twice by centrifugation in PBS and transferred to sterile tubes for storage at −80°C until further analysis.

### Two dimensional gel electrophoresis (2DE)

Isoelectric focusing was performed on Multiphor II system (Amersham GE-Health, Sweden). Briefly, 300 μg nuclear membrane protein of HCC, fibrotic liver and HepG2 cell line were dissolved in rehydration buffer (7 M urea, 2 M Thiourea, 4% CHAPS (3-[(3-cholamidopropyl) dimethylammonio]-1-propanesulfonate), 0.2% Ampholyte, 15 mM DTT (Dithiothreitol) and trace amount of bromophenol blue) and applied to IPG strips (11 cm, pH 3–10, NL) allowing to rehydrate overnight. The focusing was carried out at 20°C, following gradient change in voltage: 500 V for 1 h, gradient up to 1000 V over 1 h, gradient to 5000 V over 1 h, and focusing was continued at 5000 V for 8.5 h to give a total of 64kVh. Later the IPG strips were subjected to a two step reduction and alkylation by equilibrating the strips for 20 min in 50 mM Tris–HCl, pH 8.8, 6 M urea, 30% glycerol, 2% SDS, bromophenol blue, and 0.5% DTT, followed by another 20 min in 50 mM Tris–HCl, pH 8.8, 6 M urea, 30% glycerol, 2% SDS (sodium dodecyl sulphate), bromophenol blue and 4.5% iodoacetamide (IAA) at room temperature. Second dimension was conducted in 1 mm thick 12.5% polyacrylamide gels at 100 V for 6 h. The gels were visualized by silver staining [50], each sample were performed in triplicate. Digital images of the gels were taken by gel documentation system (BioRad, USA).

### Western blotting

Nuclear fractionated proteins (100 μg) were transferred electrophoretically (100 V for 4 hr) onto PVDF (Polyvinylidene fluoride) membrane (Amersham, GE Health, Sweden). The membranes were blocked with 5% BSA (bovine serum albumin) for 1 h at 4°C and incubated overnight with primary antibody anti- cytochrome b5A (ABCAM-UK) (1:1000 dilutions). The blots were washed three times with TBST (Tris-buffered saline with Tween) buffer and incubated for 1 hr at 4°C with goat polyclonal rabbit IgG (ABCAM-UK) (1: 5000 dilutions). Immunoblots signals were developed by chromomeric substrate-3, 3’-diaminobenzidine (DAB, Sigma-UK).

### Immunoprecipitation and 2DE (IP-2DE)

Tissue homogenates were prepared with hand homogenizer (Pyrex, Japan) by suspending the tissues in lysis buffer (7 M urea, 2 M thiourea, 0.4% CHAPS) `containing protease and phosphatase inhibitors (Roche, Germany). Sepharose G beads suspension (50 μl) (Protein-G sepharose 4 fast Flow, GE Healthcare, Life Sciences, Sweden), was centrifuged at 2000–3000 rpm for 2 min. The pellet was mixed with 450 μl HEPES (4-(2-hydroxyethyl)-1-piperazineethanesulfonic acid) buffer (50 mM HEPES pH 7.6, 200 mM NaCl, 0.4% CHAPS) and centrifuged again. The washing steps were repeated four times and HEPES buffer (450 μl) was added to the pellet and vortex again. Protein extract (0.4 mg/ml) was diluted with HEPES buffer to a final volume of 300 μl and washed protein G sepharose (200 μl) was added and incubated for 30 min at 4°C with continuous shaking. The sample was then centrifuged at 13000 rpm at 4°C for 5 min. The supernatant was incubated overnight with 5 μl of anti-S-nitroso-cysteine (SNO-Cys) antibody (ABCAM, UK) at 4°C. Activated protein G sepharose beads (140 μl) was added and mixed for 4 h at 4°C with continuous shaking and centrifuged for 2-3 min at 15000 rpm (4°C). The pellet was washed with HEPES buffer, four times and mixed with 140 μl lysis buffer (7 M urea, 2 M thiourea, 0.4% CHAPS, 1%Ampholyte) for 1 h with continuous shaking at room temperature. The sample was centrifuged at 13000 rpm at 4°C for 5 min and the pull-down was solubilized in rehydration buffer and separated by 2DE on 7 cm pH 3–10 NL immobilized pH gradients (IPG) strips. The strips were rehydrated overnight at room temperature. Isoelectric focusing was started at 500 V for 1 h, 1000 V for 1 h with gradual increase to 5000 V and kept constant for a total of 12000Vh. The gel strips equilibration and second dimension was performed as mentioned above.

### Immunoblotting using S-nitrosothiol specific antibody

Total protein extracts were also used for immunoblotting using SNO-Cys antibody. 100 μg protein was dissolved in rehydration buffer and IPG strips (7 cm, 3–10 NL) were rehydrated overnight. 2-DE was performed according to the same procedure as in section of Immuno-precipitation (IP-2D). The separated proteins were electrotransferred to PVDF membranes at 30 mA for 2 h on Wet Blot (Bio-Rad, USA) that later blocked with 5% TBST/milk for 1 h at room temperature. After blocking reaction, the blot was incubated in anti SNO-Cys antibody (1:2000) overnight at 4°C. Secondary antibody goat anti rabbit IgG HRP was applied (1:1000) for 1 h. The blots were thoroughly washed and developed with ECL (Amersham GE), detected on exposure film (Amersham GE, Sweden) and scanned with Canon flatbed scanner (Canon, UK).

### Imaging and statistical analysis

Gels were analyzed by Progenesis SameSpots v4.5 (Nonlinear Dynamic, UK) according to manufacture recommendation. Protein spots that were differentially expressed in tissue specimen and cell line were marked. Only spots altered consistently were selected for identification. Statistical analysis was performed using the SPSS statistics version 17.

### Immunofluorescence Staining

After de-paraffinization and rehydration by xylol different percentage of isopropanol, heat induced antigen retrieval was performed by immersing HCC liver section slides in a pre-heated steamer containing citrate buffer (95-100°C) for 30 min. Sections were blocked with Roti block (Roth, Karlsruhe, Germany) with 1:10 dilution, later washed with PBS (Phosphate buffer saline) and incubated with anti CYB5A antibody (ABCAM, UK) for 1 h at 40°C. After several steps washing membrane was incubated with secondary antibody, Alexa 488 anti-rabbit (ScantaCruze, California) for 30 min. Another slide of same samples were incubated with anti SNO-Cys antibody for 1 hr at 40°C followed by incubation with secondary antibody, Alexa 488 anti-rabbit for 1 h while nuclei were stained with DAPI for 2 min. Microscopic examination was performed on Eclipse TE2000E epi-flourescence microscope (Nikon, Japan). Images were acquired by DS-Qi1 processed using NIS-Elements software (Nikon Japan).

### Protein identification by electrospray ionization quadrupole time of flight tandem mass spectrometry (ESI-QTOF MS/MS)

Peptide analyses were carried out on an ESI-QTOF-tandem MS system (Waters) and in-gel digestion was performed as described [51] with slight modification. Briefly, gel slices were destained with the mixture of 15 mM K_3_Fe (CN)_6_ and 50 mM Na_2_S_2_O_3_, washed with deionized water and dehydrated with ACN. The spots were incubated with 100 mM ammonium bicarbonate, washed again and vacuum dried. Proteins were in-gel digested with sequencing grade modified trypsin (The slices were rehydrated for digestion with 40 μl trypsin (10 ng/ μl in 100 mM ammonium bicarbonate, pH 7.4; Promega, Mannhein, Germany) for 45 min. Excess trypsin solution was removed and the volume replaced with 100 mM ammonium bicarbonate without trypsin) overnight at 37°C. Tryptic peptides were extracted with 50% ACN/ 0.1% TFA with moderate sonication for 15 min. The extracted solutions were pooled, vacuum dried and re-dissolved in 0.1% TFA followed by injecting to the Q-TOF Ultima Global mass spectrometer (Micromass, Manchester, UK) as described before [52].

The data were acquired with the MassLynx (v 4.0) software on a Windows NT PC and further processed using ProteinLynx Global Server (PLGS, v 2.2, Micromass, Manchester, UK) as PKL (peak list) under the following settings; Electrospray, centroid 80% with minimum peak width 4 channel, noise reduction 10%, Savitzky-Golay, MSMS, medium deisotoping with 3% threshold, no noise reduction and no smoothing. The peak lists were searched using the online MASCOT (http://www.matrixscience.com) algorithm against the SwissProt 55.5 (389046 sequences; 139778124 residues).

The data were retrieved against the whole database with search parameters set as follows: enzyme, trypsin; allowance of up to one missed cleavage peptide; mass tolerance ±0.5 Da and MS/MS tolerance ±0.5 Da; modifications of cysteine carboamidomethylation and methionine oxidation when appropriate with auto hits allowed only significant hits to be reported.

### Protein data Set and pathway enrichment analysis

Swiss-Prot accession numbers of identified differentially expressed membrane proteins, retrieved by MASCOT were used to investigate similarities in protein expression alterations between stages. To investigate enrichment of specific pathways in the altered/PTMed protein expression data set, “WEB-based GEneSeTAnaLysis Toolkit” (WEBGESTALT) at http://bioinfo.vanderbilt.edu/webgestalt/ was used [53]. Protein expression changes are not isolated events, we therefore hypothesized that differentially expressed CYB5A protein may interact with others and play a role in oncogenesis. STRING 8.3 (http://string-db.org/) was used to explore the biological associations among the differentially expressed proteins [54].

## Abbreviations

HCC: Hepatocellular carcinoma; ESI-Q-TOF MS/MS: Electrospray ionization quadrupole time of flight tandem mass spectrometry; CYB5A: Cytochrome b5A; ATPD: ATP synthase subunit delta; HBB: Hemoglobin subunit beta; KEGG: Kyoto encyclopedia of genes and genomes; iNOS: Nitric oxide synthase; PTM: Post translational modification; FIBB: Fibrinogen beta chain; QCR1: Cytochrome b-c1 complex subunit 1; CYB4R3: Cytochrome b5 reductase 3; CYP17A1: Cytochrome P450 family 17, subfamily A, polypeptide 1; CYP3A5: Cytochrome P450 family 3, subfamily A, polypeptide 5; FADS2: Fatty acid desaturase 2; SCD: Stearoyl-CoA desaturase; ACSL: Acyl-CoA synthetase; NO: Nitric oxide.

## Competing interests

The authors declare that they have no competing interests.

## Authors’ contributions

NA and MAR designed the study, experiments, acquisition of data and finalized the manuscript. RK performed all experimental work (two dimensional electrophoresis, Immunohistochemistry, western blotting, MS), data analysis, interpretation and draft of the manuscript. YJYW, JF, ABAK and AMN provided samples and clinical data. AZ provided lab facility. SZ involved in S-nitrosylation technique development. All authors read and approved the final manuscript.

## Supplementary Material

Additional file 1 Expression graphs and MS/MS of differentially expressed proteins in HCC along with peptide sequence identified. Click here for file

Additional file 2 Microscope and Camera Setting. Click here for file
