# Supplementary material for: Protein expression profiling of nuclear membrane protein reveals potential biomarker of human hepatocellular carcinoma
Source: Clin Proteomics. 2013 Jun 1;10(1):6. doi: 10.1186/1559-0275-10-6 (PMC3691657; doi:10.1186/1559-0275-10-6)
Supplement: Additional file 1 — Expression graphs and MS/MS of differentially expressed proteins in HCC along with peptide sequence identified. [file 1559-0275-10-6-S1.docx]

| Spot  ID | Protein  Name | Abb | Location | Accession # | pI | M.wt | Peptide matches | Score | %age cov | Sequences |
| --- | --- | --- | --- | --- | --- | --- | --- | --- | --- | --- |
| N17 | Cytochrome b5 | **CYB5A** |  | P00167 | 4.8 | 15 | 11 | **176** | 42 | **1** MAEQSDEAVK **YYTLEEIQK**H NHSKSTWLIL HHKVYDLTK**F LEEHPGGEEV**  **51 LREQAGGDAT ENFEDVGHST DAR**EMSK**TFI IGELHPDDRP K**LNKPPETLI  **101** TTIDSSSSWW TNWVIPAISA VAVALMYRLY MAED  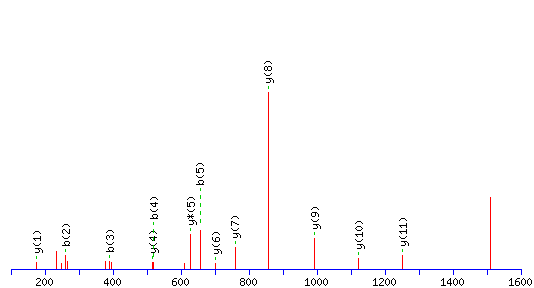 |
| N18 | ATP synthase subunit delta | **ATPD** | Mitrocondria | P30049 | 5.3 | 17 | 5 | 58 | 17 | **1** MLPAALLRRP GLGRLVRHAR AYAEAAAAPA AASGPNQMSF TFASPTQVFF  **51** NGANVRQVDV PTLTGAFGIL AAHVPTLQVL RPGLVVVHAE DGTTSKYFVS  **101** SGSIAVNADS SVQLLAEEAV TLDMLDLGAA KANLEK**AQAE LVGTADEATR**  **151 AEIQIRIEAN EALVK**ALE  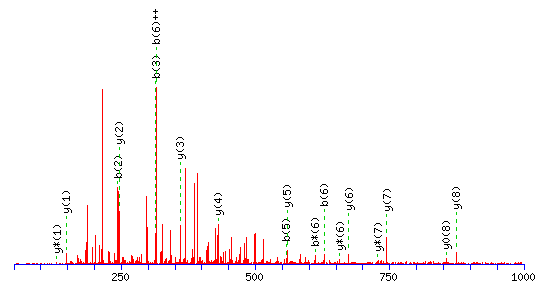 |
| N62 | **Fibrinogen beta chain** | **FIBB** | P02675 |  | 8.5 | 55 | 27 | 1009 | 41 | **1** MKRMVSWSFH KLKTMKHLLL LLLCVFLVKS QGVNDNEEGF FSARGHRPLD  **51** KKR**EEAPSLR PAPPPISGGG YR**ARPAKAAA TQKKVERKAP DAGGCLHADP  **101** DLGVLCPTGC QLQEALLQQE RPIRNSVDEL NNNVEAVSQT SSSSFQYMYL  **151** LKDLWQKRQK QVKDNENVVN EYSSELEKHQ LYIDETVNSN IPTNLRVLR**S**  **201 ILENLR**SKIQ K**LESDVSAQM EYCRTPCTVS CNIPVVSGKE CEEIIRKGGE**  **251 TSEMYLIQPD SSVKPYR**VYC DMNTENGGWT VIQNR**QDGSV DFGR**KWDPYK  **301** **QGFGNVATNT DGKNYCGLPG EYWLGNDK**IS QLTR**MGPTEL LIEMEDWK**GD  **351** KVK**AHYGGFT VQNEANK**YQI SVNKYRGTAG NALMDGASQL MGENRTMTIH  **401** NGMFFSTYDR **DNDGWLTSDP R**KQCSK**EDGG GWWYNR**CHAA NPNGR**YYWGG**  **451 QYTWDMAKHG TDDGVVWMNW K**GSWYSMRKM SMK**IRPFFPQ Q**  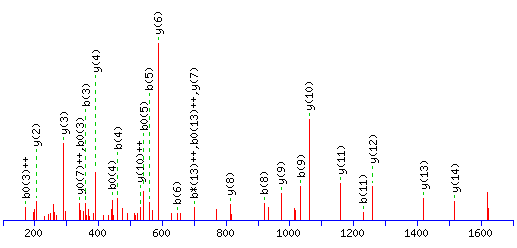 |
| N88 | **ATP synthase subunit beta** | **ATPB** | P06576 |  | 5.26 | 56 | 18 | 670 | 29 | **1** MLGFVGRVAA APASGALRRL TPSASLPPAQ LLLRAAPTAV HPVRDYAAQT  **51** SPSPKAGAAT GRIVAVIGAV VDVQFDEGLP PILNALEVQG RETR**LVLEVA**  **101 QHLGESTVRT IAMDGTEGLV R**GQK**VLDSGA PIKIPVGPET LGRIMNVIGE**  **151 PIDERGPIK**T KQFAPIHAEA PEFMEMSVEQ EILVTGIK**VV DLLAPYAK**GG  **201** K**IGLFGGAGV GKTVLIMELI NNVAK**AHGGY SVFAGVGERT REGNDLYHEM  **251** IESGVINLKD ATSKVALVYG QMNEPPGARA R**VALTGLTVA EYFR**DQEGQD  **301** VLLFIDNIFR **FTQAGSEVSA LLGR**IPSAVG YQPTLATDMG TMQERITTTK  **351** KGSITSVQAI YVPADDLTDP APATTFAHLD ATTVLSR**AIA ELGIYPAVDP**  **401 LDSTSRIMDP NIVGSEHYDV AR**GVQKILQD YKSLQDIIAI LGMDELSEED  **451** KLTVSRARKI QRFLSQPFQV AEVFTGHMGK LVPLKETIKG FQQILAGEYD  **501** HLPEQAFYMV GPIEEAVAKA DKLAEEHSS  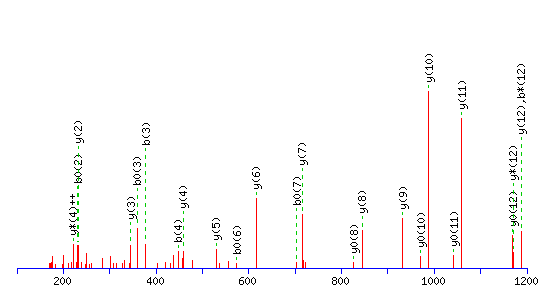 |
| N89 | **Cytochrome b-c1 complex subunit 1** | **QCR1** | P31930 |  | 5.9 | 53 | 10 | 149 | 15 | **1** MAASVVCRAA TAGAQVLLRA RRSPALLRTP ALRSTATFAQ ALQFVPETQV  **51** SLLDNGLRVA SEQSSQPTCT VGVWIDVGSR FETEK**NNGAG YFLEHLAFK**G  **101** TK**NRPGSALE K**EVESMGAHL NAYSTREHTA YYIKALSKDL PKAVELLGDI  **151** VQNCSLEDSQ IEKERDVILR EMQENDASMR DVVFNYLHAT AFQGTPLAQA  **201** VEGPSENVRK LSR**ADLTEYL STHYK**APR**MV LAAAGGVEHQ QLLDLAQK**HL  **251** GGIPWTYAED AVPTLTPCRF TGSEIRHRDD ALPFAHVAIA VEGPGWASPD  **301** NVALQVANAI IGHYDCTYGG GVHLSSPLAS GAVANKLCQS FQTFSICYAE  **351** TGLLGAHFVC DRMKIDDMMF VLQGQWMRLC TSATESEVAR GKNILRNALV  **401** SHLDGTTPVC EDIGR**SLLTY GR**RIPLAEWE SR**IAEVDASV VR**EICSKYIY  **451** DQCPAVAGYG PIEQLPDYNR IRSGMFWLRF  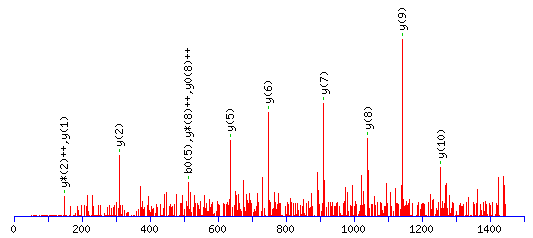 |
| N6 | Hemoglobin subunit beta | HBB | Q549N7 |  | 6.75 | 15 | 22 | 484 | 61 | \| **1** \| M**VHLTPEEKS** \| **AVTALWGKVN** \| **VDEVGGEALG** \| **RLLVVYPWTQ** \| **R**FFESFGDLS \| \| --- \| --- \| --- \| --- \| --- \| --- \| \| **51** \| TPDAVMGNPK \| VKAHGKK**VLG** \| **AFSDGLAHLD** \| **NLK**GTFATLS \| ELHCDK**LHVD** \| \| **101** \| **PENFR**LLGNV \| LVCVLAHHFG \| K**EFTPPVQAA** \| **YQKVVAGVAN** \| **ALAHKYH** \|   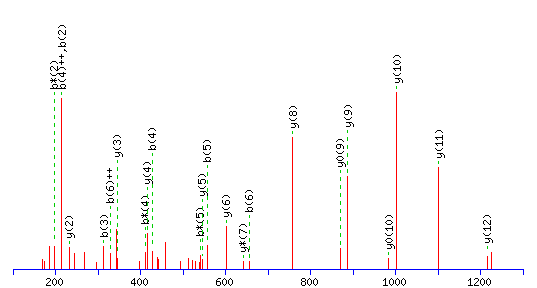 |
