# Supplementary material for: Protein expression profiling of nuclear membrane protein reveals potential biomarker of human hepatocellular carcinoma
Source: Clin Proteomics. 2013 Jun 1;10(1):6. doi: 10.1186/1559-0275-10-6 (PMC3691657; doi:10.1186/1559-0275-10-6)
Supplement: Additional file 2 — Microscope and Camera Setting. [file 1559-0275-10-6-S2.docx]

**Supplementary Data II related to microscope and camera setting**

**DIC**

Calibration= 0.8

Optics = Plan Apochromat 10x

Description= Camera Name: DXM1200c Color Camera

Numerical Aperture: 0.45

Refractive Index: 1

Camera Settings: ExposureTime: 24 ms

ExposureCompensation: 0.0

Gain: 0

Gamma: 0.450

Capturing= DXM1200c Color Camera

ExposureTime: 24 ms

ExposureCompensation: 0.0

Gain: 0

Gamma: 0.450

Offset: 0.000

KeepAE: 0

CCDCooling: 1

**DIPC**

Capturing= DXM1200c Color Camera

ExposureTime: 333 ms

ExposureCompensation: 0.0

Gain: 0

Gamma: 0.450

Offset: 0.000

KeepAE: 0

**FITCI**

Capturing=

DXM1200c Color Camera

ExposureTime: 833 ms

ExposureCompensation: 0.0

Gain: 0

Gamma: 0.450

Offset: 0.000

KeepAE: 0

**TEXAR**

Capturing=

DXM1200c Color Camera

ExposureTime: 750 ms

ExposureCompensation: 0.0

Gain: 0

Gamma: 0.450

Offset: 0.000

KeepAE: 0

CCDCooling: 0
